# Knowledge and beliefs about vaccination in pregnant women before and during the COVID-19 pandemic

**DOI:** 10.3389/fpubh.2022.903557

**Published:** 2022-08-04

**Authors:** Stefania Bruno, Lorenza Nachira, Leonardo Villani, Viria Beccia, Andrea Di Pilla, Domenico Pascucci, Gianluigi Quaranta, Brigida Carducci, Antonietta Spadea, Gianfranco Damiani, Antonio Lanzone, Bruno Federico, Patrizia Laurenti

**Affiliations:** ^1^Women, Children and Public Health Sciences Department, Fondazione Policlinico Universitario A. Gemelli IRCCS, Rome, Italy; ^2^Section of Hygiene, University Department of Life Sciences and Public Health, Università Cattolica del Sacro Cuore, Rome, Italy; ^3^Medical Oncology, Comprehensive Cancer Center, Fondazione Policlinico Universitario A. Gemelli IRCCS, Università Cattolica del Sacro Cuore, Rome, Italy; ^4^Clinical Governance, Fondazione Policlinico Universitario A. Gemelli IRCCS, Rome, Italy; ^5^Italy Local Health Authority, ASL ROMA 1, Rome, Italy; ^6^Department of Human Sciences, Society and Health, University of Cassino and Southern Lazio, Cassino, Frosinone, Italy

**Keywords:** pregnancy, vaccination, knowledge, COVID-19, vaccine hesitancy

## Abstract

**Introduction:**

Vaccine hesitancy threatens the health of populations and challenges Public Health professionals. Strategies to reduce it aim to improve people's risk perception about vaccine-preventable diseases, fill knowledge gaps about vaccines and increase trust in healthcare providers. During pregnancy, educational interventions can provide a proper knowledge about safety and efficacy of maternal and childhood vaccinations. Fighting hesitancy and clarifying doubts is fundamental during the COVID-19 pandemic, which may have affected people's knowledge and beliefs toward vaccination. This study aimed at assessing if the advent of the pandemic was associated with changes in pregnant women's knowledge and beliefs toward vaccination, and trust in healthcare services.

**Methods:**

A repeated cross-sectional study was conducted through self-reported questionnaires in a Roman teaching hospital, where educational classes about vaccinations are routinely held as part of a birthing preparation course. Data were collected on a sample of pregnant women before and during the pandemic. Free-of-charge flu vaccinations were offered to all course participants and adherence to flu vaccination was assessed.

**Results:**

The proportion of pregnant women reporting that vaccines have mild side effects and that are sufficiently tested increased from 78.6 to 92.0% (*p* = 0.001) and from 79.4 to 93.2% (*p* = 0.001), respectively. There was a reduction from 33.0 to 23.3% (*p* = 0.065) in the proportion of those declaring that healthcare workers (HCWs) give information only on the benefits and not on the risks of vaccines, and a reduction from 27.3 to 12.1% (*p* = 0.001) in those reporting that vaccines are an imposition and not a free choice of mothers. Trust in National Health Service (NHS) operators slightly decreased. Among participants, the monthly flu vaccination adherence ranged from 50.0% in November to 29.2% January for 2019–20 flu season, and from 56.3% in September to 14.5% in January for 2020–21 flu season, showing a higher vaccination acceptance in the earlier months of 2020-21 flu season.

**Conclusions:**

The pandemic may have positively affected pregnant women's knowledge and opinions about vaccinations and trust in HCWs, despite a possible negative impact on their perceptions about NHS operators. This should inspire Public Health professionals to rethink their role as health communicators.

## Introduction

Vaccine preventable diseases (VPDs) are a serious Public Health concern: their global incidence and mortality have certainly declined since vaccination programs became available, with an estimated 4–5 million deaths prevented each year (1); nonetheless, the spread of most VPDs can only be contained (and hopefully reduced) by maintaining high immunization rates over time (1). In 2017, after a large measles outbreak linked to a serious decrease in vaccination coverage, the Italian government enacted a law which extended the number of mandatory vaccines from four to ten for the age group 0–16 years (2). Although mandatory vaccination has proven useful to increase immunization coverage, it may not be helpful in reducing vaccine hesitancy, which still represents one of the greatest challenges for Public Health professionals (3–5). Vaccine hesitancy is the reluctance or refusal to vaccinate despite the availability of vaccines as defined by the World Health Organization (WHO) (3) and it is a very complex and vaccine-specific issue (6). In a recent Italian survey, parents reported safety concerns as the main reason for vaccine refusal (7), while another study showed that 19% of the participants believed that vaccines were harmful and 10% did not trust the scientific community with regard to vaccines (8). Risk perception, knowledge gaps and trust in health care providers are indeed important determinants in vaccine acceptance and should be thoroughly examined when planning vaccination strategies (9). The current Severe Acute Respiratory Syndrome Coronavirus 2 (SARS-CoV-2) pandemic may have affected vaccine hesitancy of the population at two different levels. Firstly, it exacerbated the need for an effective solution to the hesitancy, both because vaccination is a powerful tool to reduce Coronavirus Disease 2019 (COVID-19) spread (10), and because the risk of VPD outbreaks has grown in the meantime (11, 12). Indeed, attempts to limit SARS-CoV-2 contagion have led to the disruption of routine vaccination programs in many countries, expanding the already existing susceptible population of unvaccinated children (11, 12). This prompted the WHO to publish a special guide to help countries make decisions regarding the continuing provision of routine immunization services (13, 14).

Secondly, the COVID-19 pandemic forced most people to experience the urgency of vaccination, given the highly contagious nature of the new virus. It seems reasonable to expect a deep change in people's perceptions regarding infectious diseases and risk prevention, opinions on political and health institutions and approach to information. Some studies suggested a positive impact in this regard: for example, flu vaccination uptake (15, 16) and trust in political institutions (17) significantly increased during the pandemic.

Since the effects of the pandemic on vaccine hesitancy will depend mostly on the quality of the communication between lay people and Public Health professionals (4, 5), it is essential to investigate changes in people's knowledge and compliance toward vaccination.

Pregnant women represent a pivotal population group to reach given the importance of children's immunization: developing effective strategies to engage them in this practice would have the double advantage of influencing their health choices with regard to both their children and themselves. Pregnant women are indeed particularly vulnerable to developing complications from many diseases, with severe consequences also for their fetuses (18, 19) and thus they represent an important target for vaccination against VPDs (20, 21).

For these reasons, since the 2019–2020 flu season the Fondazione Policlinico Universitario Agostino Gemelli IRCCS (FPG) in Rome, Italy, has implemented an educational program about vaccination in the context of a birthing preparation course (22). By comparing questionnaires administered to course participants in both seasons (from October 2019 to January 2020 and from September 2020 to January 2021), this study aims to assess if pregnant women's vaccination awareness changed with the COVID-19 pandemic. It is hypothesized that the pandemic reduced vaccine hesitancy in this group (4, 15, 23, 24); a recent survey in Turkey showed that pregnant women's vaccine hesitancy decreased during the pandemic (25), although data were collected through a questionnaire administered only at one point in time, based on the women's recollection of their opinions before the pandemic. More specifically, this study aims at assessing the impact of COVID-19 pandemic on pregnant women's:

- knowledge and beliefs toward vaccination and use of information sources;
- trust in healthcare suppliers;
- acceptance of flu vaccination offer.

## Materials and Methods

### Study design and timeframe

A repeated cross-sectional study was conducted in the FPG teaching hospital during the periods corresponding to two flu epidemic seasons, running from October 2019 to January 2020 and from September 2020 to January 2021. The second period began 1 month earlier than the first one due to the co-circulation of flu virus and SARS-CoV-2 and the anticipation of the flu vaccination campaign in Italy in 2020 (21). The methodology used is in accordance with the most recent Guidelines for Observational Studies, STROBE (Strengthening the Reporting of Observational Studies in Epidemiology) (26).

### Study sample and setting

The study involved a convenience sample, represented by pregnant women attending the birthing preparation courses that took place at the FPG during the two observation periods. The courses were organized by the Obstetrics and Obstetric Pathology Unit of the Department of Women's and Children's Health and Public Health, and they were offered to pregnant women from the 4^th^ month of pregnancy, as well as their partners. All women who attended the courses and consented to the study were included. Each birthing preparation course consisted of six educational sessions distributed over the course of 1 month, covering subjects such as Obstetrics, Neonatology, Dental Hygiene and Public Health; courses were repeated every month for different groups of participants.

### Questionnaire and data collection

For the purposes of our study, we asked women who attended the vaccination educational session to answer an anonymous questionnaire before the session, investigating knowledge and beliefs about vaccination, perceived usefulness of various information sources and trust in institutions and healthcare workers (HCWs). The questionnaire was previously validated in a multi-centric Italian study (27, 28). Pregnant women and their partners were also given the opportunity to receive flu vaccination free-of-charge at FPG.

Due to the outbreak of SARS-CoV-2 pandemic and the subsequent declaration of a state of emergency by the Italian government in January 2020 (29), there were substantial differences in data collection methods in the two seasons. In 2019–2020 all vaccination educational sessions were held at the FPG, and questionnaires were handed out to participants; on the contrary, sessions were delivered through an online meeting platform and questionnaires were administered as online forms in 2020–2021. Moreover, in 2019–2020 on-site vaccination was offered at the end of the educational sessions, whereas in the following year the flu vaccination needed to be booked online in advance.

### Data analysis

Categorical variables were described in terms of relative frequencies (percentages). The usefulness of different information sources was assessed on a scale ranging from 1 (not useful at all) to 5 (very useful): in this case the mean value of perceived usefulness of each source was rank ordered. In order to assess differences between the two periods (before and during the pandemics), chi-square test was performed, setting statistical significance at *p* = 0.05. All statistical analyses were carried out using the software “Stata 16.” (Stata Corp, Lakeway, USA).

Finally, vaccination compliance of pregnant women was obtained by dividing the number of pregnant women who participated in the course and got vaccinated at FPG by the total number of pregnant participants. Since no information was collected if they got flu vaccination elsewhere, this denominator may also include some course participants to whom the vaccine was administered in other facilities. Vaccination compliance of their partners was obtained by dividing the number of vaccinated partners by their total number, which was equal to that of the women attending the course. The same consideration about the missing information on vaccination status applies to men.

### Ethical statement

This study is compliant with the Local Ethical Committee Standards of the FPG. It was approved and registered (Prot. N° 38264/19 ID: 2782) and was carried out in accordance with the Helsinki Declaration and EU Regulation 2016/679 (GDPR). For this kind of study, the Ethical Committee required the need for participant active consent.

## Results

### Socio-demographic and clinical characteristics of pregnant women

During the season 2019–2020, 119 pregnant women attended the course and 104 of them answered the questionnaire, with a response rate of 87.4%. During the season 2020–2021, 317 pregnant women attended the (online) course and 241 answered the questionnaire, with a response rate of 76.0%. Table 1 reports information about citizenship, marital status, educational level, employment, age and trimester of pregnancy of the sample for each season. Participant's characteristics were very similar in the two periods, as all *p-*values were > 0.05.

**Table 1:** Socio-demographic and clinical characteristics of pregnant women who answered the questionnaire before and during the COVID-19 pandemic, in the flu season 2019–20 and 2020–21.

**Variables**	**2019–20** **(*n* = 104)** **%**	**2020–21** **(*n* = 241)** **%**	***p*-value**
**Demographic and educational**			
Italian citizenship	95.2	97.5	0.254
Married	99.0	99.2	0.907
Graduate	77.9	79.2	0.789
**Occupation**			
Employee	49.0	54.8	0.206
Self-employed	23.1	13.0	
Healthcare worker	13.5	13.8	
Other Occupation	14.4	18.4	
**Pregnancy characteristics**			
First Pregnancy	96.2	92.5	0.206
Third Trimester	86.5	90.9	0.227
**Age categories**			
<30 years old	14.0	14.2	0.840
30–34 years old	41.0	44.2	
>34 years old	45.0	41.7	

### Knowledge and beliefs about vaccines before and during the COVID-19 pandemic

The percentage of women who thought that vaccines have mild side effects increased from 78.6 to 92.0% (*p* = 0.001) from the 2019–20 to the 2020–21 season. Similarly, there was a significant increase in the belief that vaccines are sufficiently tested before being placed on the market (from 79.4 to 93.2%, *p* = 0.001) (Table 2). In addition, there was a slight increase in the proportion of respondents who believed that the vaccination calendar is well designed in order to protect children (from 79.2 to 88.1%, *p* = 0.112). On the other hand, percentages were very similar in the two periods for the remaining 4 statements.

**Table 2:** Knowledge and beliefs about vaccines before and during the COVID-19 pandemic in the flu seasons 2019–20 and 2020–21.

**Claims**	2019–20 %*	**2020–21** %*	***p*-value**
Vaccines prevent potentially deadly diseases	93.7	91.5	0.643
Benefits of vaccines outweigh the risks	83.3	86.6	0.637
Most vaccines have mild side effects	78.6	92.0	0.001
Vaccines are sufficiently tested before being placed on the market	79.4	93.2	0.001
Vaccination calendar is designed to protect children	79.2	88.1	0.112
Vaccinating your own child protects other children as well	86.6	89.4	0.548
If vaccination programs were stopped, many diseases that are now very rare could come back into circulation	90.7	89.3	0.678

* *Percentage of women who answered “True”*.

### Trust in healthcare workers and the NHS and opinion on compulsory vaccination for school enrolment, before and during the COVID-19 pandemic

Table 3 reports the change in trust between the seasons 2019–20 and 2020–21 of pregnant women in HCWs and the NHS. In particular, there was a decrease in the trust about the information provided by healthcare providers (from 98.1 to 94.9%, *p* = 0.183) and a decrease from 11.2% to 7.1% (*p* = 0.215) in the percentage of women who believed that NHS workers have an economic interest in childhood vaccinations. There was also a reduction from 33.0 % to 23.3 % (*p* = 0.065) in the proportion of those declaring that healthcare workers (HCWs) give information only on the benefits and not on the risks of vaccines as well as a significant reduction in the percentage of women who believed that vaccines are an imposition and not a free choice of mothers (from 27.3 to 12.1%, *p* = 0.001). Finally, the approval for compulsory vaccination for school enrollment remained unchanged.

**Table 3:** Trust in Healthcare workers and the National Health Service (NHS) and Opinions on compulsory vaccination for school enrolment, before and during the COVID-19 pandemic in the flu seasons 2019–20 and 2020–21.

	**2019–20 %**	**2020–21** **%**	***p*-value**
Trust in Healthcare workers and the National Health Service (NHS)*			
I believe in the information provided by healthcare providers	98.1	94.9	0.183
NHS workers are prepared and updated on vaccinations	95.1	92.2	0.343
I have more trust in providers outside the NHS	10.9	17.9	0.110
NHS workers have economic interest in childhood vaccinations	11.2	7.1	0.215
NHS operators give information only on the benefits and not on the risks of vaccines	33.0	23.3	0.065
Opinions on compulsory vaccination for school enrolment**			
Vaccines are an imposition and not a free choice of mothers	27.3	12.1	0.001
I am in favor of compulsory vaccination for school enrolment	96.0	95.8	0.919

* *Percentage of women who answered “quite” or “strongly”*.

** *Percentage of women who answered “YES”*.

### Perception of the usefulness of different information sources, before and during the COVID-19 pandemic

In both seasons, the most trusted information sources were institutional sources and healthcare providers (gynecologists, pediatricians, primary care physicians, institutional sites) (Table 4). There were no changes in the ranking of the perceived usefulness of information sources during the pandemic, except for non-institutional websites, which moved from eighth to fifth position. Autonomous search for information increased from 52.9 to 65.7% (*p* = 0.025) (data not shown).

**Table 4:** Perception of the usefulness of different information sources, before and during the COVID-19 pandemic in the flu seasons 2019–20 and 2020–21.

**Information sources**	2019–20 Mean*(Ranking)	**2020–21** Mean*(Ranking)
Birthing preparation course	3.71 (1)	3.10 (1)
Gynecologist	3.21 (2)	2.88 (2)
Institutional websites	3.11 (3)	2.76 (3)
Word of mouth–friends–acquaintances	2.65 (4)	2.66 (4)
Local Health Authority/Ministry of Health information brochures	2.52 (5)	2.26 (6)
Pediatrician	2.32 (6)	2.14 (7)
Vaccination clinic	2.21 (7)	2.10 (8)
Non-institutional websites	2.19 (8)	2.30 (5)
Mass Media (i.e., TV, radio)	2.06 (9)	1.99 (9)
Trusted physician outside the NHS	2.04 (10)	1.89 (11)
General practitioner	2.00 (11)	1.97 (10)
Mobile applications	1.47 (13)	1.53 (13)
Associations against vaccinations	1.27 (14)	1.25 (14)
Other	1.65 (12)	1.60 (12)

* *Mean of perceived usefulness, measured on a scale from 1 (not useful at all) to 5 (very useful)*.

### Number of course participants and flu shots by month before and during the COVID-19 pandemic

In 2019–20, overall vaccination compliance among pregnant women was significantly higher than in 2020–21 (40.3 and 27.8%, respectively, *p* = 0.012), while the difference was not significant among their partners (32.8 and 28.1%, respectively, *p* = 0.337). Significant differences emerged in some months. During the season 2019–20, we observed a high percentage of vaccination in November (50% of the total number of women attending the course in that period), and a subsequent decrease in December and January. In the 2020–21 season, 88 women (27.8%) were vaccinated. Higher rates of vaccination adherence were observed at the beginning of the campaign (September−56.3%) with a subsequent reduction in the following months (Table 5).

**Table 5:** Number and percentages of vaccinated subjects by month before and during the COVID-19 pandemic in the flu seasons 2019–20 and 2020–21.

**Month**	**2019–20**			**2020–21**		
	**Vaccinated**		**Women attending the course *N***	**Vaccinated**		**Women attending the course *N***
	**Pregnant Women *N* (%)**	**Partners *N* (%)**		**Pregnant Women *N* (%)**	**Partners *N* (%)**	
Sept.	-	-	-	40 (56.3%)	36 (50.7%)	71*
Oct.	10 (35.7%)	6 (21.4%)	28	12 (27.9%)	15 (34.9%)	43
Nov.	22 (50.0%)	14 (31.8%)	44	12 (22.2%) ^+^	13 (24.1%)	54
Dec.	9 (39.1%)	11 (47.8%)	23	15 (17.2%)^§^	18 (20.7%) ^+^	87
Jan.	7 (29.2%)	8 (33.3%)	24	9 (14.5%)	7 (11.3%)^§^	62
Total	48 (40.3%)	39 (32.8%)	119	88 (27.8%)^§^	89 (28.1%)	317

* *Subjects who attended the course in September 2020 were vaccinated in October*.

§ *0.01 < p-value < 0.05 (comparison between same month, same group in the two periods)*.

+ *p-value < 0.01 (comparison between same month, same group in the two periods)*.

## Discussion

In our study, we found a significant increase of course participants' knowledge about vaccination from 2019–20 to 2020–21, while a decrease in their trust in NHS operators was observed.

### Knowledge and beliefs toward vaccination

The answers related to knowledge and beliefs toward vaccination showed an improvement in 2020–21 compared to the previous year. Statistically significant changes involved the topics of vaccines side effects and clinical research, showing decreased fear and increased trust in vaccine safety. Similar results were obtained in a recent study of the Italian general population (30), which showed an overall increase in trust in vaccinations between May 2020 and May 2021, especially about the importance, trustworthiness, and safety of vaccines. The results of both these surveys suggest a positive trend in vaccine confidence which may be linked to the pandemic outbreak, as also indicated by the Vaccine Confidence Project report (31). This positive trend is in contrast with the results of Palamenghi et al. (32), that found a decrease of trust in research and vaccines in the general population between the first two phases of the pandemic. Of note is the fact that although a great proportion of pregnant women were concerned about the safety of vaccines (33) before the pandemic, we observed an increase in vaccine confidence.

### Trust in healthcare workers and the national health service and opinions on compulsory vaccination for school enrollment

With regard to trust in healthcare workers, no statistically significant variations were found before and during the pandemic, nonetheless, changes in percentages may highlight issues worth investigating: the results show a percentage decrease of pregnant women who agree with the statement that NHS workers have economic interests in childhood vaccinations, and that they only provide information only about the benefits and not the risks of vaccines.

Another interesting issue involves the percentage of respondents who declared to have more trust in providers outside the NHS, which increased from 10.9% in 2019–20 to 17.9% in 2020–21. This could indicate a possible impact of the COVID-19 pandemic on the choice of healthcare professionals, which shifted from NHS providers to private sector providers. Considering opinions on mandatory vaccination, a decrease was observed in the percentage of respondents who considered vaccines an imposition (from 27.3 to 12.1%), while the agreement with obligation remained stable at 96.0%: a similar trend was found by Domnich et al. (30) among the general population between May 2020 and May 2021. These results, if confirmed by studies involving larger samples, may suggest that pregnant women's perceptions and attitudes toward NHS workers worsened during the pandemic outbreak, because of the often unsatisfactory answers given to the community needs by the NHS. Rosso et al. (34) discovered that a perceived higher quality of the NHS was strongly associated with higher levels of knowledge about vaccines in pregnant women whereas other studies found that people's relationship with healthcare services significantly changed during the pandemic. At the end of 2020, trust in HCWs decreased in a large sample of the Italian population, possibly because of communication deficits (35). Another factor of distrust might be the delaying of medical services due to the provider's decisions, particularly outpatient visits, dental visits or screening procedures, which affected large portions of population during the pandemic (36).

### Perception of the usefulness of different information sources

Regarding information sources, our results show that mean perceived usefulness of non-institutional websites increased (rising from 8^th^ to 5^th^ ranking position), even if institutional sources remained stable in a higher position. On the one hand, this figure is reassuring because the information on vaccinations from institutional websites is associated with pregnant women's greater degree of knowledge about this topic (34) but on the other hand it represents a wake-up call about the increased usage of non-institutional website, where misinformation is very common (37). The perception of the usefulness of sources did not vary significantly, with birthing preparation course and gynecologist being the first and second highest ranking in both years. Autonomous searching for information increased from 52.9 to 65.7%, which is in agreement with the increased need of information on vaccination, highlighted by Domnich et al. (30) in the general population, and with the widespread difficulties in finding “reliable and trustworthy information about the virus and its effects” reported by the Edelman Trust Barometer (38). The overall ranking of information sources preferred by pregnant women does not reflect the one described before the pandemic by Rosso et al. (33) in their systematic review, which found only one study (out of 16 studies) reporting healthcare professionals (especially midwives and GPs) as the most highly accessed resource, while other studies indicated the internet or media as mostly consulted (39). This is probably due to the differences existing between the socio-economic and cultural context of this study and that of other studies, which involved many different countries across the world. Nonetheless, there is accordance with some findings of the Italian studies, which reported word-of-mouth and non-institutional websites as very common information sources (28, 33, 34, 40).

Understanding the way pregnant women obtain information about vaccination is extremely relevant, given the association of specific sources with different levels of knowledge (33) and, in particular, the positive influence of information received from HCWs on their immunization choices (41). This is especially crucial considering the great availability of misleading information. In Chinese pregnant women, this appears to be a major issue mostly for the highly educated, who are more likely to refuse vaccination than less educated ones, probably because of larger access to information sources (42). This situation is opposite to the Italian one, where higher levels of education are significant determinants of the intention to vaccinate (34). In both contexts, offering accurate and trusted information is fundamental.

### Acceptance of flu vaccination offer

Acceptance of vaccination offer among pregnant women attending the preparation course and their partners differed in the two seasons. In 2019–20, overall vaccination compliance was significantly higher than in 2020–21 among pregnant women. This could be related to the implementation of on-site vaccination in 2019–20, before the COVID-19 pandemic. Several studies show that on-site vaccination increases vaccination compliance, both in pregnant women and their partners (43–45), and in other populations, e.g. HCWs (46, 47). Each month, a different percentage of course participants accepted the vaccination offer. In 2019–20, the highest was registered in November for pregnant women (50.0%) and December for their partners (47.8%). In 2020–21, the highest percentages were registered in September (56.3% of pregnant women and 50.7% of their partners), with a gradual decrease in the following months.

This difference in adherence by month between the two seasons may be due to the different epidemiological situations: since the COVID-19 pandemic outbreak, recommendations to get flu vaccination have been stronger and earlier, especially for at-risk categories. This may have led pregnant women and their partners to promptly accept vaccination offer at the beginning of 2020–21 flu season; in the following months, there were probably higher proportions of course participants who were already vaccinated at the time of the course vaccination offer, having received flu vaccination elsewhere. Unfortunately, data regarding course participants' vaccination status (if flu vaccine was administered elsewhere) was missing in the current study. Nevertheless, some findings show an increased willingness to receive influenza vaccination during the pandemic, in both pregnant women (42) and Italian general population (30), suggesting a higher awareness regarding this topic. This situation differs from other countries, such as Israel, where the second and third waves of COVID-19 left pregnant women's approach toward flu vaccination unchanged (48).

### Study limitations

This study has some limitations: firstly, the sample is not representative of the wider variety of Italian women who can get pregnant, because it is composed of attendants to a birthing preparation course offered in one large hospital in Rome; those who attend a birthing preparation course have generally a higher interest and awareness on their health status than pregnant women who do not.

Secondly, the course delivery in the 2020–21 season, which was carried on an online meeting platform, may have negatively affected both response to the questionnaire and adherence to vaccination offer. The advantage was that more people could attend the course, being connected to an Internet platform from home. Thirdly, the response rate in 2020–21 decreased compared to the previous year (from 87.4 to 76.0%), probably due to the different way of questionnaire administration (49, 50); possible confounders, as availability of Internet access and ability to use it, were not studied. Nonetheless, the percentage of respondents was higher if compared to other online and email surveys (51).

## Conclusion

Our study results suggest that the pandemic may have positively affected pregnant women's knowledge and opinions about vaccinations, and highlights a decreasing trust toward the Italian NHS. To guide the population of pregnant women through the misleading and often worrying information available, the role of Public Health professionals should be emphasized and re-examined so they can organize effective health communication programs.

## Data Availability Statement

The raw data supporting the conclusions of this article will be made available by the authors, without undue reservation.

## Ethics Statement

The studies involving human participants were reviewed and approved by Institutional Review Board (or Ethics Committee) of Fondazione Policlinico Universitario Agostino Gemelli IRCCS. The patients/participants provided their written informed consent to participate in this study.

## Author contributions

SB, PL, GD, AD, BC, BF, and AL contributed to the study conception and design. Material preparation and data collection were performed by SB, LN, VB, AD, LV, and DP. LN, LV, and BF performed the statistical analysis. The first draft of the manuscript was written by SB, LN, LV, and BF. SB, GQ, BC, AS, GD, AL, BF, PL, and DP commented on the latest version of the manuscript. SB, AL, BF, BC, GF, and PL supervised the study. All authors contributed to the article and approved the submitted version.

## Conflict of interest

The authors declare that the research was conducted in the absence of any commercial or financial relationships that could be construed as a potential conflict of interest.

## Publisher's note

All claims expressed in this article are solely those of the authors and do not necessarily represent those of their affiliated organizations, or those of the publisher, the editors and the reviewers. Any product that may be evaluated in this article, or claim that may be made by its manufacturer, is not guaranteed or endorsed by the publisher.
